# Occupational Therapy Services for Community-Dwelling Patients With Stroke in Thailand: Explanatory Sequential Mixed Methods Study

**DOI:** 10.2196/94765

**Published:** 2026-05-01

**Authors:** Sopida Apichai, Jananya P Dhippayom, Pachpilai Chaiwong, Savitree Thummasorn, Piyawat Trevittaya, Waranya Chingchit

**Affiliations:** 1 Department of Occupational Therapy Faculty of Associated Medical Sciences Chiang Mai University Muang Chiang Mai, Chiang Mai Thailand

**Keywords:** stroke, occupational therapy, rehabilitation, community, barriers, social participation, quality of life, qualitative study, mixed methods

## Abstract

**Background:**

Stroke is a major cause of long-term disability, and upper extremity (UE) impairment frequently limits independence in activities of daily living (ADLs) and community participation. In Thailand, although acute stroke care has improved, continuity of rehabilitation in community settings remains uneven. Currently, there is limited empirical evidence identifying occupational therapy (OT) services and the barriers affecting service delivery in practice.

**Objective:**

This study aimed to examine current OT practices among community-dwelling patients with stroke in Thailand, identify barriers affecting service delivery, and explore therapists’ perspectives on the future development of community-based rehabilitation.

**Methods:**

An explanatory sequential mixed methods design was used. Phase I (survey phase) involved an online survey of 59 occupational therapists to describe service patterns and perceived barriers, while phase II (interview phase) comprised semistructured interviews with 7 experienced occupational therapists to obtain deeper insight into practice contexts and challenges. Quantitative data were analyzed descriptively, and qualitative data were analyzed using thematic analysis.

**Results:**

OT services were primarily individualized and focused on functional activities, particularly basic ADLs (49/59, 83.05%), cognitive (44/59, 74.58%), and sensorimotor training (40/59, 67.80%). Service frequency was generally limited, and nonstandardized assessments were commonly used alongside policy-required measures, such as the Barthel Index. Major barriers were identified at organization/leadership, client, and therapist levels, including resource shortages (50/59, 84.75%), inaccessible to service (45/59, 76.27%), lack of public awareness about OT (45/59, 76.27%), and lack of time (42/59, 71.19%). Occupational therapists described adapting practice through client-centered and culturally responsive approaches, with active caregiver involvement. A proposed home-based stroke rehabilitation box set emerged as a potential strategy to support continuity of rehabilitation.

**Conclusions:**

Community-based OT in Thailand operates within structural constraints, while maintaining occupation-centered practice. Strengthening services may require system-level support and practical strategies to enhance continuity of care.

## Introduction

Stroke remains a leading cause of long-term disability both in Thailand and worldwide [1,2]. Although improvements in acute management have enhanced survival rates, many patients with stroke continue to experience persistent functional limitations that affect daily life [3,4]. In particular, upper extremity (UE) dysfunction is common and often long-lasting, restricting independence in self-care, work-related tasks, and community participation [5]. Limitations in activities of daily living (ADLs) are closely associated with reduced quality of life and diminished social engagement [6]. For many individuals, meaningful recovery depends not only on early medical treatment but also on sustained rehabilitation within the home and community environment.

In Thailand, considerable progress has been made in strengthening acute stroke pathways; however, continuity of rehabilitation remains uneven [2,7]. The length of hospital stay is typically short, averaging between 3 and 7 days [7-10], and only a proportion of patients with stroke receive structured inpatient rehabilitation prior to discharge [7,11]. As a consequence, many patients are discharged with ongoing impairments and variable access to follow-up rehabilitation services [12]. The introduction of the Intermediate Care (IMC) policy by the Thailand Ministry of Public Health sought to address this gap by promoting transitional and community-based rehabilitation through multidisciplinary collaboration [13]. Although evaluations of IMC programs report improvements in functional outcomes [11,14,15], implementation at the service level continues to face practical challenges. These include resource limitations, administrative demands, transportation barriers, and uneven service distribution across regions [16]. Such challenges are not unique to Thailand but are characteristic of rehabilitation systems operating within constrained resource environments.

Occupational therapists play a crucial role in supporting patients with stroke in regaining functional independence and participation. Through occupation-based assessment and intervention, occupational therapists address not only motor recovery but also the integration of meaningful activities into daily routines and life roles [17,18]. In a community setting, practice extends beyond impairment-focused approaches and requires an in-depth understanding of broader environmental, cultural, and social contexts [19-22]. In Thailand, where family caregiving plays a prominent role and community resources vary widely, culturally responsive and context-sensitive practice becomes especially important [23,24]. However, despite the recognized importance of community-based occupational therapy (OT), there remains a limitation of empirical research evidence describing how these services are delivered in practice. Much of the existing literature focuses on program outcomes or policy implementation, while less attention has been given to therapists’ day-to-day experiences, service structures, and the barriers they encounter when working with community-dwelling patients with stroke. A clearer understanding of current OT practice patterns and contextual challenges is necessary to inform sustainable service development. Exploring therapists’ perspectives may provide insight into how rehabilitation is adapted within existing policy frameworks and resource realities. It may also reveal locally grounded innovations that can strengthen continuity of care beyond clinical settings. Therefore, the aim of this study was to examine current OT practices for stroke rehabilitation among community-dwelling patients with stroke in Thailand, identify key barriers affecting service delivery, and explore occupational therapists’ perspectives on future development of community-based rehabilitation services. By integrating findings from a nationwide survey with in-depth qualitative interviews, this study sought to contribute contextually grounded evidence to inform the improvement of community stroke rehabilitation in Thailand.

## Methods

### Study Design

This study used an explanatory sequential mixed methods design consisting of two phases [25]. Phase I (survey phase) was a cross-sectional online survey designed to collect quantitative data on existing OT practices and barriers in community-based OT services for Thai patients with stroke living in the community. Phase II (interview phase) used a generic qualitative design to build upon the initial findings. Through semistructured interviews, this phase gathered qualitative insights regarding service experiences, barriers to practices, and occupational therapists’ perspective on developing effective OT services in community stroke rehabilitation. The explanatory sequential mixed methods design enhanced the comprehensiveness of the findings and allowed qualitative data from the descriptive qualitative design to clarify and expand upon the quantitative results [26].

### Participants

#### Phase I: Survey Phase

Participants were recruited using purposive sampling from the official registry of the Occupational Therapist Association of Thailand (OTAT), which contained 1171 registered occupational therapists at the time of the study. To ensure relevance to the study objectives, participants were required to (1) be registered occupational therapists in Thailand, (2) have at least 1 year of experience working with community-dwelling patients with stroke, and (3) confirm their willingness to participate. In total, 75 occupational therapists who met these criteria were formally invited to participate in the online survey via OT social media networks.

#### Phase II: Interview Phase

A selective sampling strategy was used to identify key informants based on their primary practice setting and expertise in providing community-based stroke rehabilitation. This approach aimed to reflect a range of service contexts and professional perspectives. Inclusion criteria for the interview phase were (1) at least 3 years of experience delivering services to community-dwelling patients with stroke, (2) active provision of community stroke rehabilitation at least twice per week, and (3) willingness to provide informed consent for audio-recorded interviews. Due to the anonymous nature of the survey phase, participants for the qualitative phase were recruited independently of the survey respondents. Initial key informants were purposively selected based on their recognized expertise and primary practice setting in providing community-based stroke rehabilitation. Subsequently, the snowball sampling technique was used, wherein initial participants identified other practitioners with significant experience in providing OT services for community-dwelling patients with stroke in Thailand to ensure a comprehensive range of professional insights. Recruitment and interviews continued until data saturation was reached. Saturation was assessed through a concurrent analysis process, where interviews were transcribed and coded in tandem with ongoing recruitment. The researchers monitored for thematic redundancy, specifically looking for the point where no additional insights or new themes emerged from subsequent interviews [27]. Given that the participants were expert key informants with extensive experience in community-based stroke rehabilitation, a high degree of conceptual density was reached. Recruitment was concluded when the last two consecutive interviews confirmed that the existing thematic framework was stable and sufficiently addressed all research questions.

### Research Instruments

Given the contextual characteristics of community OT practice in Thailand and the lack of a validated instrument specific to this topic, the authors developed the research instruments. Instrument development was informed by a review of the relevant literature on community rehabilitation, stroke recovery, and OT practice.

In the survey phase, a questionnaire consisting of two sections was used. The first section of the questionnaire consisted of 13 items designed to collect demographic and practice-related information. These items included personal demographics (sex, education level, and employment status) and clinical practice characteristics (eg, primary practice setting, years of experience in community-based stroke rehabilitation, average number of stroke patients per week, frequency and duration of services, intervention formats, and the recovery stages of stroke patients served, categorized by Brunnstrom stages). The second section focused on perceived barriers to rehabilitation services for community-dwelling patients with stroke. This section also included open-ended questions to gather participants’ views on the characteristics of an effective community-based rehabilitation program. A combination of dichotomous, multiple-response, and open-ended questions was used to gather both quantitative trends and qualitative insights.

For the interview phase, a semistructured interview guide was developed to explore therapists’ experiences in service delivery, practical challenges encountered in community settings, and perspectives on future development of OT services. The interview questions are presented in Textbox 1.

**Questions in the semistructured interview guide.**

1. Please introduce yourself (educational background, work history, and experience in providing services to Thai patients with stroke living in the community).

2. What is the form or nature of providing services to patients with stroke with limited arm and hand movement living in your community?

3. What do you think of the form of occupational therapy (OT) for patients with stroke living in the community with limited arm and hand movement in Thailand from the past to the present?

4. What do you think should be the form of OT rehabilitation for this group of patients?

5. What do you think are the barriers to OT rehabilitation for this group of patients?

6. How do you think these barriers should be eliminated?

7. What do you think will help promote OT rehabilitation for this group of patients to be more effective?

### Instrument Validation

The developed instruments were evaluated for content validity and clarity by three experienced occupational therapists, each with more than 5 years of experience in stroke rehabilitation. They independently reviewed all surveys and interview guide questions. The Index of Item-Objective Congruence (IOC) was used to assess the alignment of each item with the study objectives. Items with IOC scores between 0.50 and 1.00 were retained, while items scoring below 0.50 were revised or removed [28]. In addition, a pilot test was conducted with five occupational therapists. Their feedback was used to refine the wording, clarity, and practicality of the instruments.

### Ethical Considerations

Ethical approval was obtained from the Ethical Review Committee for Human Research, Faculty of Associated Medical Sciences (approval #161/2562). All procedures complied with national ethical guidelines for research involving human participants [29].

In the survey phase, eligible participants received detailed information about the study and the informed consent process. The online survey in this study was reported in accordance with the CHERRIES (Checklist for Reporting Results of Internet E-Surveys) checklist [30].

In the interview phase, participants’ consent was obtained to audio-record interviews and take field notes to capture contextual details. Written informed consent was obtained prior to the interviews.

### Data Collection

In the survey phase, eligible participants were provided with the study objectives and procedures, together with a link to the online survey distributed via OT social media networks. Reminder messages were sent to eligible participants 1 week and 3 days before survey closure to increase response rates and reduce nonresponse bias [31]. In the interview phase, in-depth interviews were conducted either face to face or via online videoconferencing, depending on participant preferences and logistical considerations. Each interview lasted approximately 30-45 minutes.

The data collection was divided into two distinct periods:

- Phase I (survey phase): conducted from February 2020 to December 2021. This phase captured the initial impact of the COVID-19 pandemic on OT services.
- Phase II (interview phase): conducted from January 2022 to April 2025. This phase focused on long-term adaptations and current challenges in the postpandemic landscape.

### Data Analysis

#### Quantitative Analysis

In the survey phase, survey responses were exported from Google Forms and analyzed using Microsoft Excel. Descriptive statistics, including frequencies and percentages, were used to summarize demographic characteristics, practice patterns, and perceived barriers. Responses to open-ended questions were analyzed using content analysis [32], in which responses were grouped inductively into thematic categories based on recurring patterns and shared meanings.

#### Qualitative Analysis

In the interview phase, interview recordings were transcribed verbatim. Thematic analysis was conducted following the six-phase framework by Braun and Clarke [33]. This trustworthy process included (1) familiarization with the data, (2) generation of initial codes, (3) searching for themes, (4) reviewing themes, (5) defining and naming themes, and (6) writing the report. Two researchers (authors SA and WC) independently conducted the initial coding using both inductive and deductive approaches. Discrepancies were resolved through discussion and consensus. Themes were further refined through collaborative review to ensure they accurately represented the data. Thematic analysis was completed between May 2022 and April 2025, ensuring that the integration of findings reflected the most recent professional context.

### Trustworthiness and Rigor

This study used credibility, transferability, dependability, and confirmability as strategies to ensure trustworthiness and rigor. The credibility triangulation strategy used both quantitative and qualitative data obtained from participants among various contexts to ensure accuracy of the findings. The transferability strategy of thick descriptions was used by providing detailed background information, including participants’ experiences, main practice settings, and the procedures for data collection and analysis. The confirmability strategy of peer debriefing was used by researchers to interpret results and reduce data bias [34,35].

## Results

### Quantitative Findings

The online survey was successfully completed by 59 occupational therapists, yielding a response rate of 78.67%. Table 1 shows the demographic and professional characteristics of the participants. The majority were female (35/59, 59.32%) and held a bachelor’s degree (50/59, 84.75%) in OT, while the remaining participants had postgraduate qualifications. Almost all participants (58/59, 98.30%) reported full-time employment. A substantial proportion worked in district hospitals (25/59, 42.37%), suggesting that occupational therapists in these settings are actively involved in providing community-based stroke rehabilitation services.

**Table 1:** Demographic and professional practice profiles of quantitative study participants (N=59).

Characteristics		Participants, n (%)
**Sex**		
	Female	35 (59.32)
	Male	24 (40.68)
**Highest academic qualification**		
	Bachelor’s degree	50 (84.75)
	Master’s degree	9 (15.25)
**Main practice setting**		
	Subdistrict hospital	1 (1.70)
	District hospital	25 (42.37)
	Provincial hospital	12 (20.34)
	Specialized hospital/institute	17 (28.81)
	University hospital	1 (1.70)
	Private hospital	3 (5.08)
**Employment status**		
	Full-time	58 (98.30)
	Part-time	1 (1.70)
**Experience working with community-dwelling Thai patients with stroke (years)**		
	1-2	7 (11.86)
	3-5	11 (18.64)
	6-10	14 (23.73)
	>10	27 (45.77)
**Primary Brunnstrom stage of UE^a^ recovery in patients with stroke served (ranked by frequency)**		
	1	8 (13.56)
	2	12 (20.34)
	3	20 (33.90)
	4	14 (23.73)
	5	5 (8.47)
	6	0
**Average number of patients with stroke per week**		
	1-5	19 (32.20)
	6-10	13 (22.03)
	>10	27 (45.77)
**Average number of services per case per week**		
	1 time	29 (49.15)
	2-3 times	15 (25.42)
	4-6 times	5 (8.47)
	Every day	10 (16.96)
**Average service duration per case (minutes)**		
	<30	2 (3.39)
	30-60	49 (83.05)
	>60	8 (13.56)
**Intervention forms**		
	Individual	40 (67.80)
	Group	1 (1.70)
	Both individual and group	18 (30.50)

^a^UE: upper extremity.

In terms of professional experience, nearly half of the participants (27/59, 46%) reported more than 10 years of experience working with community-dwelling patients with stroke. Most patients served by these therapists were classified as Brunnstrom Stage 3 (20/59, 34%), followed by stages 4 (14/59, 24%) and 2 (12/59, 20%), reflecting a population with moderate motor impairment. Individual therapy (40/59, 68%) was the most common mode of intervention. Many participants (27/59, 46%) reported managing more than 10 stroke cases per week. The most common service frequency per case was once per week (29/59, 49%), and the average duration of each session ranged from 30 to 60 minutes (49/59, 83%). These findings suggest a relatively high workload with limited service frequency per individual client. Nearly half of the participants had over 10 years of practice experience and managed a high weekly caseload of patients with stroke, indicating a significant depth of clinical experience of participants.

Almost all participants (36/59, 61%) allocated the majority of their time (51%-75%) to intervention rather than assessment, referral, or documentation, as shown in Table 2. Physicians (49/59, 83%) were the most frequent source of referrals, followed by nurses (20/59, 34%) and self-referrals (19/59, 32%), indicating that referral pathways remain largely a hospital-based (Table 3).

**Table 2:** Distribution of time allocation across OT^a^ service components (N=59).

Service component	Time allocation				
	Never, n (%)	<25%, n (%)	26%-50%, n (%)	51%-75%, n (%)	>75%, n (%)
Assessment	0	44 (74.57)	14 (23.73)	1 (1.70)	0
Intervention	0	0	7 (11.86)	36 (61.02)	16 (27.12)
Referral	9 (15.25)	49 (83.05)	0	1 (1.70)	0
Documentation	0	58 (98.30)	1 (1.70)	0	0

^a^OT: occupational therapy.

**Table 3:** Frequency of referrals from various sources to OT^a^ services (N=59).

Referral source	Not applicable, n (%)	Never, n (%)	Sometimes, n (%)	Often, n (%)
Physicians	0	3 (5.09)	7 (11.86)	49 (83.05)
Nurses	7 (11.86)	14 (23.73)	18 (30.50)	20 (33.91)
Occupational therapists	3 (5.08)	10 (16.95)	44 (74.58)	2 (3.39)
Self-referral	3 (5.08)	5 (8.47)	32 (54.24)	19 (32.21)
Family referral	9 (15.26)	23 (38.98)	18 (30.50)	9 (15.26)
Community health center/community agency staff	3 (5.08)	10 (16.95)	37 (62.71)	9 (15.26)
Health insurance system	13 (22.03)	31 (52.55)	11 (18.64)	4 (6.78)
Screening from the community by occupational therapists	7 (11.86)	17 (28.82)	24 (40.68)	11 (18.64)

^a^OT: occupational therapy.

Table 4 illustrates assessment tools used in community OT services according to standardized, nonstandardized, or both standardized and nonstandardized assessments. All participants (59/59, 100%) reported assessing basic activities of daily living (BADLs) and cognitive function. Sensorimotor (58/59, 98.30%), and physical (57/59, 96.61%) components were also commonly evaluated. Nonstandardized assessments were widely used across most occupational performance domains. Standardized assessments were used more frequently in the evaluation of BADLs (42/59, 71.19%) compared to other domains, suggesting greater accessibility or familiarity with formal measurement tools in this area.

**Table 4:** Characteristics of assessment instrument use across occupational performance domains (N=59).

Occupational performance domain	Standardized assessment, n (%)	Nonstandardized assessment, n (%)	Both standardized and nonstandardized assessment, n (%)	Never assessed, n (%)
BADL^a^	42 (71.19)	12 (20.34)	5 (8.47)	0
IADL^b^	25 (42.37)	23 (38.98)	8 (13.57)	3 (5.08)
Rest and sleep	2 (3.39)	38 (64.41)	0	19 (32.20)
Education	4 (6.78)	45 (76.27)	0	10 (16.95)
Work/productive activities	5 (8.47)	50 (84.75)	0	4 (6.78)
Play	4 (6.78)	39 (66.10)	0	16 (27.12)
Leisure	4 (6.78)	49 (83.05)	0	6 (10.17)
Social participation	5 (8.47)	45 (76.27)	0	9 (15.26)
Sensorimotor components	25 (42.37)	25 (42.37)	8 (13.56)	1 (1.70)
Cognitive components	29 (49.15)	21 (35.59)	9 (15.26)	0
Psychosocial and psychological components	11 (18.64)	41 (69.50)	2 (3.39)	5 (8.47)
Temporal aspects	4 (6.78)	48 (81.35)	1 (1.70)	6 (10.17)
Physical components	6 (10.17)	49 (83.05)	2 (3.39)	2 (3.39)
Social components	5 (8.47)	49 (83.05)	1 (1.70)	4 (6.78)
Cultural components	3 (5.08)	51 (86.44)	1 (1.70)	4 (6.78)

^a^BADL: basic activity of daily living.

^b^IADL: instrumental activity of daily living.

As shown in Table 5, the most frequently implemented interventions were BADL training (49/59, 83.05%), cognitive training (44/59, 74.58%), and sensorimotor training (40/59, 67.80%). These patterns correspond closely with the reported assessment priorities and reflect a significant focus on restoring BADL function and physical and cognitive functional recovery.

**Table 5:** Frequency of OT^a^ intervention types across occupational performance domains (N=59).

Occupational performance domain	Never, n (%)	Sometimes, n (%)	Often, n (%)
BADL^a^	0	10 (16.95)	49 (83.05)
IADL^b^	4 (6.78)	34 (57.63)	21 (35.59)
Rest and sleep	32 (54.24)	23 (38.98)	4 (6.78)
Education	26 (44.07)	27 (45.76)	6 (10.17)
Work/productive activities	8 (13.56)	41 (69.49)	10 (16.95)
Play	28 (47.46)	26 (44.07)	5 (8.47)
Leisure	12 (20.34)	34 (57.63)	13 (22.03)
Social participation	15 (25.42)	29 (49.16)	15 (25.42)
Sensorimotor components	3 (5.08)	16 (27.12)	40 (67.80)
Cognitive components	1 (1.70)	14 (23.72)	44 (74.58)
Psychosocial and psychological components	12 (20.34)	32 (54.24)	15 (25.42)
Temporal aspects	20 (33.90)	30 (50.85)	9 (15.25)
Physical components	5 (8.48)	27 (45.76)	27 (45.76)
Social components	14 (23.73)	28 (47.46)	17 (28.81)
Cultural components	18 (30.51)	31 (52.54)	10 (16.95)

^a^BADL: basic activity of daily living.

^b^IADL: instrumental activity of daily living.

Perceived barriers identified from survey responses were grouped into three categories: client related, occupational therapist related, and organizational/leadership related (Multimedia Appendix 1). The most frequently reported client-related barriers were inaccessibility to rehabilitation services (45/59, 76.27%) and limited public awareness of OT (45/59, 76.27%). Among therapist-related barriers, time constraints (42/59, 71.19%) were most frequently reported, indicating perceived workload pressures. Interestingly, the shortage of occupational therapists (6/59, 10.17%) was reported less frequently than other barriers. Organizational and leadership-related barriers were most commonly associated with resource shortages (50/59, 84.75%), including limited equipment, funding, and transportation.

### Qualitative Findings

Seven occupational therapists with diverse backgrounds in community rehabilitation participated in the semistructured interviews. Their experience in working with community-dwelling patients with stroke ranged from 10 to 18 years (Table 6). All participants were involved in community rehabilitation services, including the IMC program. Thematic analysis generated several key themes, which are presented next with illustrative quotations.

**Table 6:** Demographic and professional experience profiles of qualitative study participants (N=7).

Participant ID	Sex	Main practice setting	Experience working with community-dwelling Thai patients with stroke (years)
T1	Female	District hospital	10
T2	Female	District hospital	12
T3	Female	District hospital	18
T4	Female	District hospital	16
T5	Female	Subdistrict hospital	15
T6	Female	Provincial hospital	17
T7	Female	Provincial hospital	16

#### Theme 1: Occupational Therapy Service and Clinical Reasoning

The setting in which OT services were delivered influenced both the frequency and the structure of the services. In community rehabilitation settings, therapists scheduled sessions once per month over a 6-month period, consistent with the IMC protocol. For clients who were able to access outpatient services, sessions were more frequent, usually two to three times per week. For long-term patients with stroke facing geographic, financial, or motivational barriers, service frequency was often reduced, sometimes occurring only every 4-6 months. In some cases, clients were lost to follow-up due to these combined constraints.

Most services were delivered individually, with sessions lasting approximately 60 minutes. Each session generally included assessment, intervention, referral, and documentation components. Documentation was described as essential, particularly for meeting IMC key performance indicators and supporting funding requirements. These highlight the influence of administrative structures on clinical practice.

Participants described their professional reasoning as grounded in a client-centered approach, emphasizing individualized OT goals within specific environmental contexts. Although nonstandardized assessments were commonly used due to time constraints and contextual variability, the Barthel Index (BI) was routinely administered to fulfill IMC reporting requirements. This illustrates how therapists balance clinical judgment with policy-mandated procedures. As one participant clearly stated:

Most stroke cases are both chronic and acute. The first assessment is with the Barthel Index before looking at ADL. It must be done for IMC data entry and financial reimbursement. Treatment remains functionally oriented-focusing on ADL-but incorporates techniques like PNF, Rood, NDT, and weight-bearing, with activities used to promote function. If the patient has a caregiver, we train them for continuity at home.

Therapist 1

#### Theme 2: Cultural Orientation

Participants emphasized the importance of culturally responsive practice in community rehabilitation. A recurring pattern was the integration of family-centered approaches that align with Thai collectivist values. Caregiving was often framed as a shared responsibility rather than a burden. Therapists described actively supporting and empowering family members to participate in the rehabilitation process. One therapist explained:

Emphasize the importance in the beginning: Encourage, let the relatives prepare themselves that the patient is not a burden. If we train them, they will get better, and we will not be tired...enhance the confidence of the caregiver to go to see the patient at home and empower them, because the main thing is that they need to be taken care of.

Therapist 4

This approach reflects the integration of cultural awareness into therapeutic relationships and service delivery.

#### Theme 3: Task-Oriented, Individualized Interventions

Interventions were described as combining preparatory and occupation-based approaches. Therapists highlighted the importance of task-oriented training to improve functional ADL performance. Programs were tailored to the individual’s occupational roles and daily routines, reflecting client-centered practice. One therapist provided the following example:

Each case will have a different program...For example, the chicken rice seller is not yet strong in holding a knife, so we try to make the knife handle bigger. We start with weight training, then wrap putty around a steel rod to mimic his work tool. He practices cutting the putty into pieces before returning to cutting chicken, starting by preparing food for personal use first.

Therapist 4

#### Theme 4: Challenging in Developing Home-Based Rehabilitation Tools

Participants identified the need for practical home-based rehabilitation tools to support continuity of care. One therapist proposed the development of a stroke rehabilitation box set consisting of portable, low-cost equipment designed to facilitate UE functional training and enhance accessibility of rehabilitation in home and community settings.

Beyond the equipment itself, participants emphasized the importance of training caregivers and community health volunteers to use these tools effectively. This was viewed as a strategy to extend rehabilitation beyond clinical visits and reduce therapist workload.

I want to develop a set of equipment designed for a stroke box set that can be used practically and be portable—no one has done it yet. It should include safe glasses, balls of different sizes, ADL items, functional training tools, or even special equipment for hand strength and dexterity…having one box to demonstrate or rotate could really help.

Therapist 3

#### Theme 5: Factors Influencing Program Feasibility

Participants identified both external and internal factors affecting the feasibility of community-based stroke rehabilitation. External factors included policy constraints, fund shortages, and limited availability of rehabilitation equipment. Internal factors included client motivation, caregiver burden, and therapist workload

The roles of caregivers and community health volunteers were also discussed. Some caregivers received formal training and compensation through long-term care systems, while others participated voluntarily. Participants noted that structured training and ongoing professional guidance could enhance the effectiveness of both caregivers and volunteers in supporting rehabilitation, particularly in rural communities. One participant reflected:

Volunteers or caregivers can be the same person, but caregivers must have at least 70 hours of certified training. They are paid per visit. Volunteers work out of goodwill and are not paid, but many people in the community take on several roles.

Therapist 5

**Figure 1:**
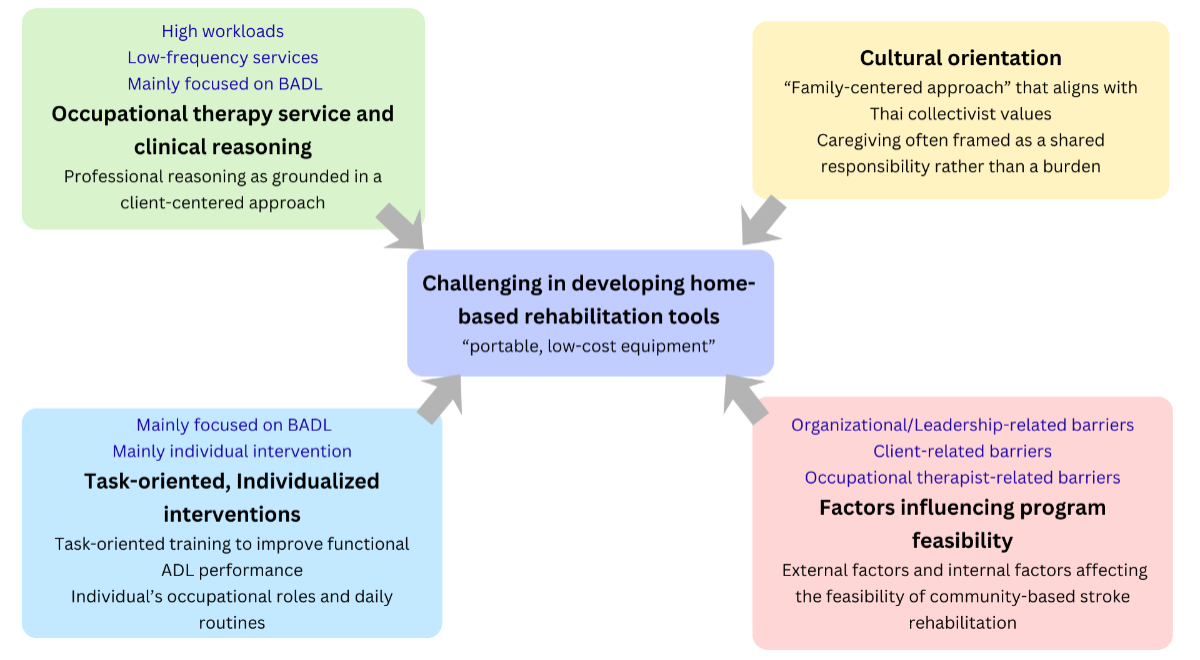
Integrated findings from quantitative and qualitative data. ADL: activity of daily living; BADL: basic activity of daily living.

The integrated findings from quantitative and qualitative data are summarized in Figure 1.

## Discussion

### Principal Findings

This study examined current OT practices among community-dwelling patients with stroke in Thailand and explored the contextual factors shaping service delivery. The findings suggest that community-based OT services are largely individualized, functionally oriented, and delivered within practical constraints that influence frequency, assessment approaches, and intervention planning.

The quantitative findings indicate that most therapists manage relatively high caseloads, while providing services at a limited frequency per client. Sessions are predominantly focused on BADL, cognitive interventions, and sensorimotor interventions, which correspond to the clinical presentation of patients with stroke commonly classified within moderate motor impairment stages. The interviews revealed that these are delivered through task-oriented training adapted to the client’s home environment. This synergy demonstrates that Thai community occupational therapists maintain occupation-based practice by embedding therapeutic goals within the practical functional needs of patients with stroke. These patterns reflect pragmatic prioritization of functional independence within available time and resource limitations. Similar trends toward function-focused, time-limited community rehabilitation have been reported in other low-, middle-, and high-income countries, where service intensity is often shaped by workforce capacity and infrastructure constraints rather than ideal clinical dosing [36-38]. The relatively frequent use of nonstandardized assessments, alongside routine administration of the BI for IMC reporting purposes, suggests that therapists balance clinical judgment with policy requirements. Rather than indicating inconsistency in practice, this pattern may represent contextual adaptation to administrative and system-level expectations.

Barriers to service delivery were reported at multiple levels. Organizational and leadership-related barriers, particularly resource shortages and transportation limitations, were most frequently identified in quantitative data. These findings highlight the structural factors that shape community rehabilitation, beyond individual therapist effort. Client-related barriers, including limited public awareness of OT and restricted access to services, further affect continuity of care. These findings are supported by our qualitative data, which elucidate the practical reality that occupational therapists experience a discharge rehabilitation gap postdischarge were geographic, financial, or motivational barriers directly hinder continuity of care. Interestingly, although workforce shortages were reported less frequently, time constraints were commonly identified, suggesting that workload pressures and administrative responsibilities may reduce opportunities for sustained therapeutic engagement. Our findings are consistent with previous studies, which have identified infrastructure, workforce, and policy challenges in Thai community OT rehabilitation services [12,39]. The qualitative narratives explain this discrepancy, revealing that therapists’ time is not necessarily consumed by a lack of colleagues but by heavy administrative burdens linked to IMC reporting and the logistical challenges of traveling between community sites. This suggests that the perceived barrier is not a lack of clinicians per setting but a lack of clinical time within the current administrative framework. A related study conducted among Thai community-dwelling patients with stroke and their family caregivers revealed that a lack of knowledge and information related to rehabilitation contributes to a major barrier to the restoration of the functional abilities of patients with stroke [40]. For these participants, education from health care professionals and social support from local and central governments in accessing rehabilitation were perceived as crucial for well-being [40]. Together, these findings indicate that strengthening community rehabilitation requires attention not only to clinical training but also to system organization and resource allocation. Previous studies have suggested that strengthening community rehabilitation systems requires integrated service models, improved referral pathways, and policy-level investment in workforce and infrastructure [40-43]. These system-level strategies may complement the practice-level adaptations identified in this study, particularly in addressing structural barriers, such as resource limitations and service accessibility. The qualitative findings provide further insight into how therapists respond to these constraints. Participants described clinical reasoning that remains grounded in client-centered and occupation-based principles, even within policy-driven environments. The influence of IMC key performance indicators was evident in documentation and assessment procedures [11]; however, therapists continued to tailor interventions according to clients’ occupational roles and daily routines. Examples of task-oriented training adapted to specific work contexts illustrate how occupation-based practice is maintained in community settings [39].

Cultural context also emerged as an important factor shaping rehabilitation processes. Therapists described actively engaging family members as partners in care and framing caregiving within culturally meaningful values. In settings where service frequency is limited, caregiver involvement becomes central to continuity of rehabilitation. This finding is consistent with the literature emphasizing caregiver-mediated rehabilitation in community stroke recovery [24,40]. In the Thai context, strong family networks may serve as a practical resource that supports functional recovery when formal services are constrained. International studies have likewise emphasized the value of caregiver-mediated interventions in sustaining home-based stroke rehabilitation, particularly where professional contact time is limited [44,45].

The COVID-19 pandemic acted as a catalyst for home-based rehabilitation in Thailand [46]. As hospital resources were diverted to the pandemic response, OT services were forced to adapt quickly to community-dwelling environments. This temporal context may have influenced the patterns observed and should be considered when interpreting the findings. This context likely intensified the barriers identified in Multimedia Appendix 1, such as resource shortages and the need for caregiver-mediated interventions, as practitioners had to rely more heavily on family support when face-to-face clinical visits were restricted.

This cultural reliance, combined with identified resource shortages, directly informed the participants’ proposal of a stroke rehabilitation box set to support home-based intervention. Although conceptual at this stage, the idea reflects therapists’ efforts to extend rehabilitation beyond clinic visits through portable, low-cost tools combined with caregiver training. Although home-based technologies offer the advantages of flexibility in location and time for rehabilitation therapy, they also allow patients to receive feedback from therapists remotely as the use of rehabilitation technologies in the home environment requires technical training [47,48]. Rather than representing a technological innovation, this proposal suggests a practical strategy for improving continuity of care within existing resource limits. Future research should examine the feasibility, acceptability, and clinical outcomes of such home-based support models.

### Limitations and Future Research

Although this study presents its strengths, certain limitations should be acknowledged. First, although the survey included participants from a range of practice settings, it may not fully represent the geographical diversity or variability of rehabilitation service structures across all regions of Thailand. Service organization and resource availability may differ in areas not captured within the sample. Second, the qualitative component included only occupational therapists. The perspectives of patients with stroke, their primary caregivers, and other rehabilitation professionals were not explored in this study. Including these stakeholders’ voices in future research would provide a more comprehensive understanding of community rehabilitation processes and challenges. Third, although the study identified practice-based ideas, such as the proposed home-based rehabilitation kit, these concepts remain exploratory and require further investigation regarding their feasibility, effectiveness, and scalability within community settings. Fourth, a notable limitation of this study is the extended 5-year data collection period, which spanned the pre- and post–COVID-19 eras. The global pandemic significantly disrupted health care systems in Thailand, potentially causing shifts in OT practice and practitioners’ perspectives over time. Although the sequential design allowed us to explore these transitions, the long duration may introduce historical bias, as participants’ responses might be influenced by systemic changes not present in 2020. This temporal context should be considered when interpreting the findings. However, a strength of this prolonged engagement is the ability to capture the resilience and evolution of OT services during one of the most challenging periods for the Thai health care system.

### Conclusion

Overall, the findings illustrate that community-based OT in Thailand operates within identifiable structural constraints, while maintaining core occupation-centered principles. Therapists demonstrate flexibility in adapting assessment and intervention strategies to local realities. Efforts to strengthen community rehabilitation should therefore address both system-level factors, such as resource allocation, transportation, and administrative workload, and practice-level supports, such as caregiver training and context-specific intervention tools.

Taken together, these findings offer a contextually grounded understanding of community-based OT practice in stroke rehabilitation, which may help inform ongoing efforts to strengthen services in similar settings.

## Appendix

Multimedia Appendix 1 Perceived barriers to community OT services. OT: occupational therapy.

## Abbreviations

ADL: activity of daily living
BADL: basic activity of daily living
BI: Barthel Index
IADL: instrumental activity of daily living
IMC: Intermediate Care
IOC: Index of Item-Objective Congruence
OT: occupational therapy
UE: upper extremity

## Data Availability

The data used in the manuscript are available from the corresponding author upon reasonable request.
