# Supplementary figures and images for: Occupational Therapy Services for Community-Dwelling Patients With Stroke in Thailand: Explanatory Sequential Mixed Methods Study
Source: J Particip Med. 2026 May 1;18:e94765. doi: 10.2196/94765 (PMC13179487; doi:10.2196/94765)

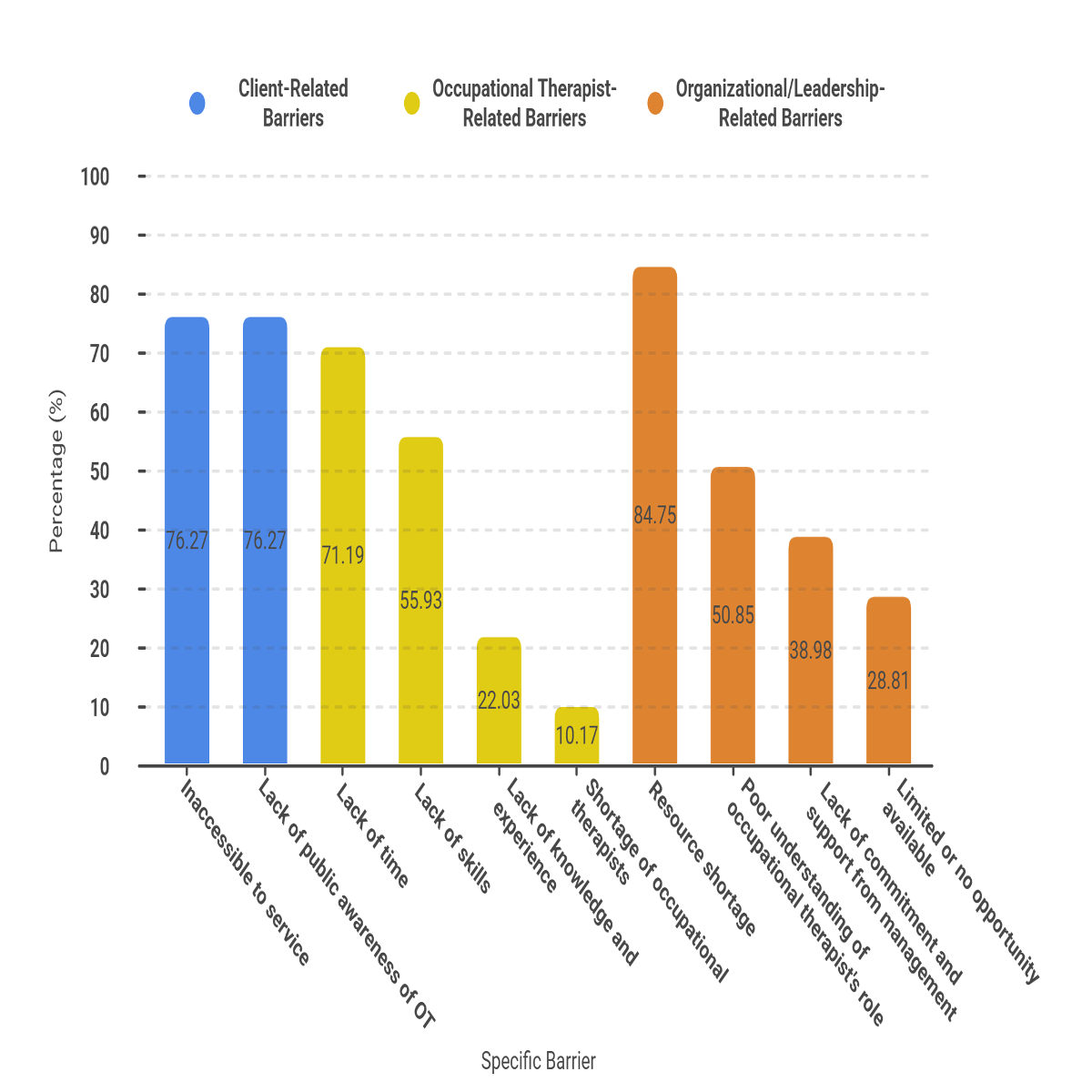

Supplement: Multimedia Appendix 1 [file jopm_v18i1e94765_app1.png]
